# Insulin Receptor-Related Receptor Activation by Artificial Double-ER Mutations in the Transmembrane Domain

**DOI:** 10.3390/ijms27104364

**Published:** 2026-05-14

**Authors:** Oxana V. Serova, Alina A. Gavrilenkova, Andrey S. Kuznetsov, Alexander S. Goryashchenko, Alexandra R. Agisheva, Yaroslav V. Bershatsky, Vladislav A. Lushpa, Olga T. Zangieva, Mikhail S. Karbyshev, Andrei S. Gerasimov, Ivan S. Okhrimenko, Roman G. Efremov, Igor E. Deyev, Eduard V. Bocharov

**Affiliations:** 1 Shemyakin–Ovchinnikov Institute of Bioorganic Chemistry, Russian Academy of Sciences, Miklukho-Maklaya Str. 16/10, 117997 Moscow, Russia; oxana.serova@gmail.com (O.V.S.); alycat1008@gmail.com (A.A.G.); andrej.kuznecov@phystech.edu (A.S.K.); asgoryash@gmail.com (A.S.G.); alexagisheva@gmail.com (A.R.A.); bershackyjaroslav@gmail.com (Y.V.B.); lushpa1696@gmail.com (V.A.L.); r-efremov@yandex.ru (R.G.E.); 2 Moscow Center of Advanced Studies, Kulakova Str. 20/1, 123592 Moscow, Russia; asgerasimoff@mail.ru (A.S.G.); i.s.okhrimenko@yandex.ru (I.S.O.); 3 Scientific Research Institute for Systems Biology and Medicine, Nauchny Proezd 18, 117246 Moscow, Russia; 4 National Medical and Surgical Center Named After N. I. Pirogov, Nizhnyaya Pervomayskaya Str. 70, 105203 Moscow, Russia; olga.dok.oz@gmail.com; 5 Research Division, Moscow Polytechnic University, Bolshaya Semyonovskaya ul. 38, 107023 Moscow, Russia; mikhail_karbyshev@hotmail.com

**Keywords:** receptor tyrosine kinases, insulin receptor family, activation, pH sensitivity, mutations, molecular dynamics, transmembrane helix–helix interactions

## Abstract

The orphan insulin receptor-related receptor (IRR), in contrast to its homologs from the insulin receptor family, is activated by a mildly alkaline extracellular medium. We have previously demonstrated that IRR activation is defined by two synergistic sites located in the dimeric extracellular domain. Here, we describe artificial mutations in the IRR transmembrane domain that promote receptor activation. First, using molecular modeling based on the NMR-derived structure, we proposed amino acid substitutions that could enhance non-covalent interactions between the transmembrane segments of the IRR dimer. These mutations were subsequently tested for effects on pH sensing by IRR. We showed that double-mutant A938E-A939R was highly phosphorylated at neutral pH and still sensitive to alkaline pH. Remarkably, the double substitution of V929E-G930R resulted in strong basal phosphorylation of the receptor over the pH titration range. Through site-directed mutagenesis, we demonstrated that the transmembrane domain plays a critical role in IRR activation, allowing for targeted control of functioning of the receptor, including its pH sensitivity.

## 1. Introduction

The insulin receptor (IR) family consists of three highly homologous receptor tyrosine kinases (RTKs): IR, insulin-like growth factor receptor (IGF-IR), and insulin receptor-related receptor (IRR). The RTK monomer molecule has a multi-domain structure and consists of three main parts: a large extracellular ligand-binding domain (ectodomain), which provides specific perception of the extracellular signal; a transmembrane (TM) α-helical domain; and a cytoplasmic domain with kinase activity, transmitting the signal farther to the intracellular cascade.

A necessary condition for the functioning of RTKs is their homo- or heterodimerization (both ligand-dependent and ligand-independent), accompanied by conformational rearrangements and the active participation of all domains to achieve a key signaling event—multiple autophosphorylation of the C-terminal portion of the receptor. In general, ligand binding activates RTKs by inducing receptor dimerization [1]. But there is evidence that a subset of RTKs forms dimers or oligomers even in the absence of an activating ligand [1,2,3]. A ligand-independent association (pre-dimerization or oligomerization) has also been found in an inactive “dormant” state in many RTK family members, including EGFR, Tie2, and Eph [3,4].

The receptors of the insulin receptor (IR) family are expressed on the cell surface as disulfide-linked nonactive homo- and heterodimers. Ligand binding (insulin or IGF-I or II) induces structural changes in the dimeric receptors that stimulate tyrosine kinase activity and cell signaling. Recent studies indicate the important role of RTK TM domains in ligand-dependent activation of the receptors. In the inactive state, the TM domains are in a conformation, which prevents the proper interaction of cytoplasmic parts of the molecule, allosterically inhibiting phosphorylation of the target Tyr residues. Upon ligand binding, conformational changes in the extracellular domain, in turn, cause a change in the mutual disposition of TM domains. As a result, intracellular tyrosine kinase domains undergo spatial rearrangement, leading to the formation of proper contacts, which permit them to phosphorylate each other, inducing a cellular response.

Though ligand-independent activation of RTKs may occur at non-physiologically high levels of expression (e.g., in cancer cells), in general, receptor–ligand binding is the main mechanism of RTK activation, with the exception of the IRR, HER2/Erbb2, and c-Met receptors. IRR and HER2/Erbb2 do not have peptide or protein ligands and can be activated by extracellular alkaline medium (pH > 7.9) [5,6]. The c-Met receptor can also be activated by alkali media, comparable to activation by HGF [7]. IRR activation triggers intracellular signaling that starts with phosphorylation of signaling adapter protein IRS-1, leading to actin cytoskeleton rearrangement in beta pancreatic MIN6 cells, where IRR is expressed endogenously [8]. Furthermore, in vivo analysis of the IRR knockout mouse phenotype under experimentally induced alkalosis revealed the role of IRR as a regulator of bicarbonate excretion in the kidney [9]. Qualitative analysis of IRR/IR chimeras showed the involvement of extracellular domains in IRR alkali sensing, primary roles played by the L1C, FnIII-2, and FnIII-3 subdomains [10,11,12]. As is inherent to the IR receptor family, the ectodomains of IRR in an inactive state adopt a symmetric Λ-shaped conformation with spatially separated TM domains, whereas upon activation, they switch to Γ- and T-shaped conformations, facilitating the appropriate dimerization of the TM domains [13,14].

Understanding the structural implications of RTK alterations is important in guiding therapeutic options. Recent clinical and biophysical studies show that the double-polar V659E-G660R mutation located inside the N-terminal dimerization motif of the HER2 RTK TM domain stabilizes the active dimeric state of the receptor through intermonomeric hydrogen bonding in the cell membrane, resulting in uncontrolled receptor activity, cell transformation, and oncogenesis [2].

Here, we present data from site-directed mutagenesis of the IRR TM domain, which switches the receptor to a constitutively active state that is insensitive or weakly affected by alkaline pH. First, using molecular modeling based on the NMR-derived structure of the IRR TM domain [15], we identified the position for double-ER substitution, analogous to the known HER2 mutation, which is capable of inducing of the strong interaction between the TM segments. Subsequently, we generated plasmids encoding the mutant IRR receptors and tested the pH sensitivity of these mutants when expressed in HEK293 cells. Hence, our data demonstrate that the TM domain is essential for IRR activation, providing a means for targeted control of the receptor’s functioning—in particular, its pH sensitivity.

## 2. Results

### 2.1. Preliminary Structures of the Wild-Type and Double-ER Mutant Dimers of the IRR TM Domain

**Figure 1:**
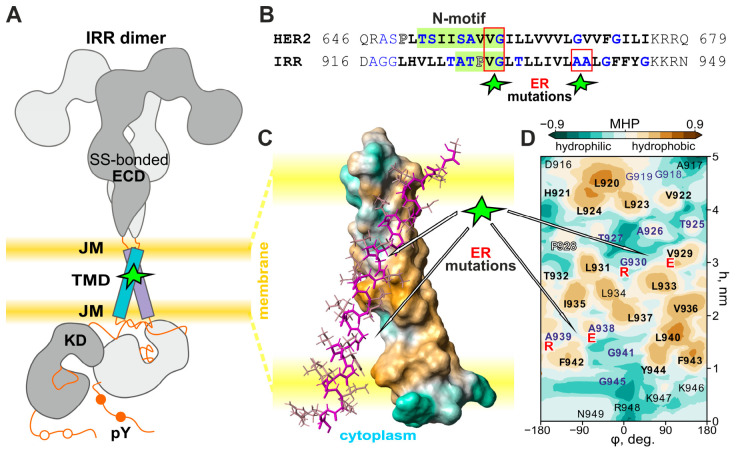
The IRR TM domain sequence, along with its presumable dimerization mode and hydrophilic–hydrophobic properties. (**A**) The model of the full-length IRR receptor in the active T-shaped conformation. ECD—ectodomain; JM—juxtamembrane region; TMD—transmembrane domain; KD—kinase domain; pY—phosphorylated tyrosine residues. (**B**) Alignment of the TM domain sequences of HER2 and IRR receptors. N-motif—HER2 TM helix dimerization GG4-like pattern corresponding to the active receptor state. The TM helix packing interfaces are usually covered by small, weakly polar side-chain residues such as G, A, S, and T (in blue), often forming GG4-like motifs characterized by small–X_3_–small tetrad repeats. The potential N-terminal dimerization GG4-like motif A^926^TPVG^930^ presumably associated with the active IRR state is highlighted in green. The positions of oncogenic ER mutation V659E-G660R of HER2 and of N- and C-terminal substitutions V929E-G930R and A938E-A939R inserted into the IRR TM domain are marked by red boxes. (**C**) Proposed dimeric structure demonstrating the association of the IRR TM domain analogously to HER2 in a right-handed α-helical bundle through the N-terminal motif, forming a weakly polar cavity on the TM helix surface. Hydrophobic and hydrophilic (polar) surfaces of one dimer subunit are shown in blue and brown, respectively, according to the values of the molecular hydrophobicity potential (MHP). (**D**) Hydrophobicity map of the wild-type IRR TM helix colored according to the arbitrary MHP units, presented in cylindrical coordinates associated with the helix. Axis values correspond to the rotation angle around the helical axis (φ) and the distance along the latter (h). ER substitution positions are highlighted in red.

Earlier, we found that both orphan IRR and HER2 RTKs can undergo pH-dependent autophosphorylation by the alkali medium. In these single-spanning membrane receptors, pH-induced conformational rearrangements of extracellular domains trigger specific dimerization of TM domains, leading to allosteric activation of cytoplasmic kinase domains and subsequent signal propagation across the membrane into the cell (Figure 1A) [5,6,15]. Clinical and biophysical studies have revealed numerous oncogenic mutations in the TM domain of HER2, which cause uncontrolled receptor activity, cell transformation, and oncogenesis [2]. In particular, it was found that polar mutations, including the double-ER substitution of V659E-G660R, which are associated with the constitutively active receptor, cluster near the N-terminal dimerization motif of the HER2 TM domain and can stabilize the active receptor dimer by formation of intermonomeric hydrogen bonds and salt bridges within the membrane [2]. Alignment of the TM domain sequences of HER2 and IRR receptors identifies the position of a double-ER substitution of V929E-G930R that could activate the IRR receptor in a manner analogous to the HER2 oncogenic mutation (Figure 1B,C). In contrast, another ER substitution, i.e., A938E-A939R, was selected in the C-terminal part of the IRR TM domain to evaluate the specificity of the N-terminal double mutation and contrast its influence on pH-dependent receptor activation pathways.

First, to study possible dimerization modes, we used the PredDimer algorithm [16] to generate a wide range of dimeric structures for the wild-type IRR TM domain and its ER-mutant forms—namely, 60 models for IRR^WT^, 49 models for IRR^MN^ (V929E-G930R double mutation), and 59 for IRR^MC^ (A938E-A939R double mutation). No significant changes were detected after reconstruction of charged termini, followed by energy minimization. However, after clustering of the resulting MD states, we significantly reduced the number of solutions. We found eight clusters for IRR^WT^, seven clusters for IRR^MN^, and nine for IRR^MC^. Four structures were outside clusters for IRR^WT^, six for IRR^MN^, and four for IRR^MC^; most of these solutions represent non-symmetrical dimers, while others are closed to clusters but beyond the cut-off used in the clustering algorithm. Notably, we observed that the corresponding clusters for the wild-type and mutant IRR TM domains exhibited fairly similar parameters across all three TM sequence types (Table S3).

### 2.2. Behavior of the Predicted Dimer Structures of the Wild-Type and Mutant IRR TM-Domain Dimer in the Explicit Lipid Bilayer

We employed MD relaxation in an explicit POPC bilayer to assess the structural stability of each possible dimeric conformation of the wild-type and mutant IRR TM domains identified through the clustering described above. Both backbone RMSD and the evolution of dimer geometry were monitored in the course of 200 ns MD trajectories (Supplementary Table S4). Comparative analysis revealed that in most cases, parallel and left-handed dimer conformations of IRR^WT^ exhibited slightly greater stability than right-handed ones. The introduction of the ER pairs into the hydrophobic region stabilizes structures in which the highly polar and oppositely charged glutamate and arginine residues are located at the dimerization interface, whereas other configurations become less stable. Nevertheless, the simulations revealed a range of possible structures for each IRR TM-domain form, making it impossible to unambiguously identify the definitive dimeric model in any case. Based on the MHP distribution analysis and stability estimation (Figure S1), a parallel and polar interface is preferred for IRR^WT^, whereas the mutants exhibit a tendency to form crossed structures with ER pairs at the interface. Representative dimeric structures of the wild-type and mutant IRR TM domains are presented in Figure S2. Notably, the observed redistribution of preferred conformations and interaction interfaces implies that the ER substitutions can modulate protein oligomerization.

### 2.3. The Impact of the Single- and Double-Point ER Substitutions on the pH-Sensitive Properties of IRR

In order to verify the impact of the ER substitutions on the TM domain structure and their role in pH-dependent activation of the IRR receptor, we generated several mutant forms of IRR containing corresponding single- and double-point substitutions. HEK293 cells were transfected with the plasmids encoding mutant forms of IRR with the double-ER substitutions V929E-G930R and A938E-A939R, as well as the corresponding single mutations, i.e., V929E, G930R and A938E, A939R.

**Figure 2:**
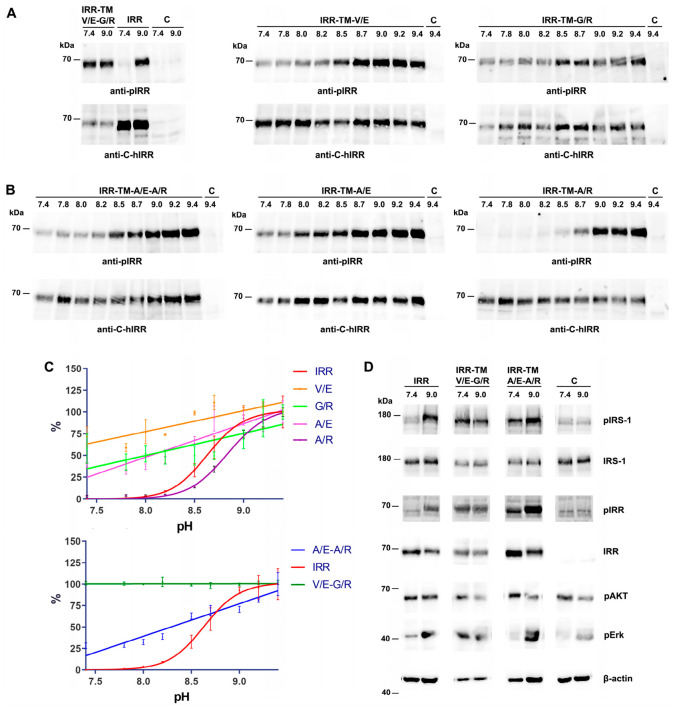
The impact of single and double mutations at positions V929E, G930R and A938E, A939R in the transmembrane domain of IRR on receptor activation. (**A**) and (**B**) HEK293 cells were incubated in F-12 medium at the indicated pH values, lysed, and analyzed by Western blotting using antibodies to phosphorylated (pIRR) and total (C-hIRR) forms of IRR. (**C**) Comparative quantitative analysis of pH-dependent activation of IRR and its double mutants (V/E-G/R and A/E-A/R, bottom panel) alongside the corresponding single mutants (V/E, G/R, A/E, and A/R, upper panel). Phosphorylation signals were quantified and normalized according to the anti-C-hIRR signals. Normalized signals were plotted vs pH of the tested solutions. Values are means ± SEM (*n* = 4). (**D**) Western blot analysis of the effect of double mutations V/E-G/R and A/E-A/R on phosphorylation of the intracellular signaling adapter protein IRS-1. HEK293 cells were incubated in F-12 medium at pH 7.4 or 9.0, and analyzed by Western blotting with antibodies to phosphorylated IRR (pIRR), total IRR (IRR), phosphorylated IRS-1 (pIRS-1), total IRS-1 (IRS-1), phosphorylated AKT (pAKT), phosphorylated Erk (pErk) and β-actin (β-actin). C–control untransfected HEK293 cells.

Using Western blot analysis with antibodies against the phosphorylated and total forms of IRR, we found that the double V929E-G930R substitution led to strong basal receptor autophosphorylation at a pH of 7.4 (Figure 2A,C). Furthermore, alkaline treatment up to a pH of 9.4 had no effect on the autophosphorylation level (pIRR/IRR ratio) of the V929E-G930R double mutant, whereas the wild-type IRR showed a significant response. Corresponding single substitutions (V929E and G930R) also induced basal receptor phosphorylation at a pH of 7.4. In contrast to the double mutant, however, these single IRR mutants exhibited modest activation in response to increasing pH. Western blot analysis reveals revealed linear pH dependence for the mutants, in contrast to the S-shaped curve observed for the wild-type IRR activation. Notably, both the single A938E and double A938E-A939R mutants of IRR demonstrated measurable phosphorylation, even at neutral pH, while preserving its inherent pH-dependent regulatory properties (Figure 2B,C). The pH-dependent activation profile of the single A939R mutant closely resembles that of the wild-type IRR, displaying an S-shaped response curve shifted by ~0.2 pH units toward more alkaline values.

It is known that IRR receptor activation leads to phosphorylation of the intracellular substrate protein IRS-1, an essential mediator of intracellular signaling [5]. Accordingly, we assessed the capacity of the IRR double mutant forms to activate downstream signaling cascades in cells. We stained lysates of HEK293 cells expressing the wild-type IRR receptor, and its mutant forms V929E-G930R and A938E-A939R with antibodies against the phosphorylated and total forms of the IRS-1 protein (Figure. 2D). We found that the wild-type IRR receptor induced IRS-1 activation only upon alkaline stimulation, whereas V929E-G930R and A938E-A939R mutants exhibited basal IRS-1 phosphorylation already at neutral pH, suggesting a gain-of-function phenotype. To further characterize downstream signaling, we assessed the phosphorylation status of AKT and Erk. Both the V929E-G930R and A938E-A939R mutants specifically promoted Erk phosphorylation, while AKT phosphorylation remained unaffected (Figure 2D). 

**Figure 3:**
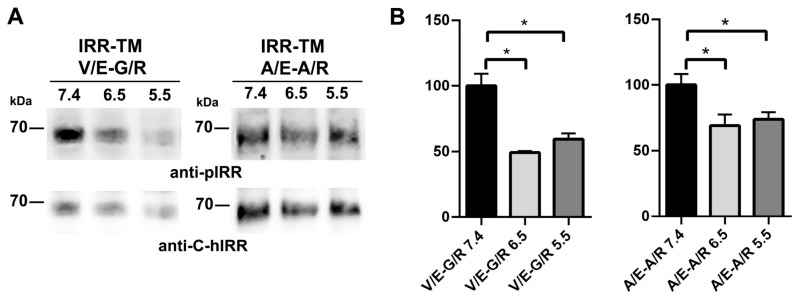
The effect of the N- and C-terminal double mutations V929E-G930R and A938E-A939R on IRR phosphorylation under acidic conditions. (**A**) HEK293 cells expressing IRR mutant forms with substitutions V/E-G/R and A/E-A/R were incubated in F-12 medium at pH 7.4, 6.5, or 5.5., and analyzed by Western blotting with anti-pIRR and anti-C-hIRR antibodies. (**B**) Quantitative analysis of pH-dependent activation of IRR double mutants V/E-G/R (left panel) and A/E-A/R (right panel). Phosphorylation signals (pIRR) were quantified and normalized to the corresponding total IRR levels (anti-C-hIRR). Normalized signals were plotted as a function of pH. Data represent mean ± SEM (*n* ≥ 4). Asterisks indicate statistically significant differences (* *p* < 0.05).

Thus, the combined modeling data and receptor activation assays suggest that the V929E-G930R and A938E-A939R double mutants exhibit non-covalent interactions between their TM domains, thereby inducing constitutive receptor activation under neutral and mildly alkaline conditions (at pH values above 7.4). Further measurements conducted under acidic conditions (pH 5.5–6.5) revealed that both double mutants exhibited a decreased basal phosphorylation. Specifically, the level of phosphorylation of the V929E-G930R mutant was reduced by half, while the A938E-A939R mutant showed a ~30% decline (Figure 3).

## 3. Discussion

Over the past few years, several important findings have shed light on structural changes within the insulin receptor family upon activation by different agonists. Cryo-EM structures of the insulin receptor (IR), insulin-like growth factor 1 receptor (IGF-1R), and insulin receptor-related receptor (IRR) in their active states have revealed new details of conformational changes of their ectodomains within the receptor complex following activation by insulin, IGF-1, or alkali (in the case of IRR) [14,17]. It is noteworthy that, unlike other family members, only the IRR ectodomain structure has been structurally characterized by Cryo-EM in both basal and active states, suggesting that the IR and IGF-1R ectodomains may adopt multiple conformations in the unbound state.

The prevailing paradigm of the activation of RTK of the insulin receptor family involves a conformational transition from an inactive Λ-shaped form to a T-shaped form of a disulfide-linked ectodomain dimer upon ligand binding. In the Λ-like conformation, the TM domains are spatially separated, and their dimerization is difficult. Upon transition to the T-shaped state, the TM domains approach each other and become capable of direct interaction through N-terminal dimerization motifs [15,18]. In the IGF-1R–insulin complex, a distinct asymmetrical Γ-shaped (drop-like) conformation has been observed, where the TM domains are also positioned to facilitate their dimerization. The alkali-induced conformation transitions from the inactive Λ-shaped to the active Γ- and T-shaped conformations of IRR have also been detected by high-resolution atomic force microscopy [13,19].

Although the RTK TM domains do not interact with ligands directly, they play an important role in receptor activation. Based on structural-dynamic studies of various RTK TM domains, we previously proposed a lipid-mediated mechanism implying that signal transduction through the membrane and allosteric regulation of RTK are inclusively mediated by coupled protein–protein and protein–lipid interactions, particularly in transmembrane and juxtamembrane regions [3]. Recently, using NMR spectroscopy, we elucidated the structural-dynamic properties of the TM domains of IR, IGF-1R, and IRR and suggested a unique molecular mechanism of receptor activation distinct in some details from other RTKs—in particular, by bending in the N-terminal part of the TM helix mediated by a flexible P-hinge proximal to the conserved intramembrane proline residue [15].

**Figure 4:**
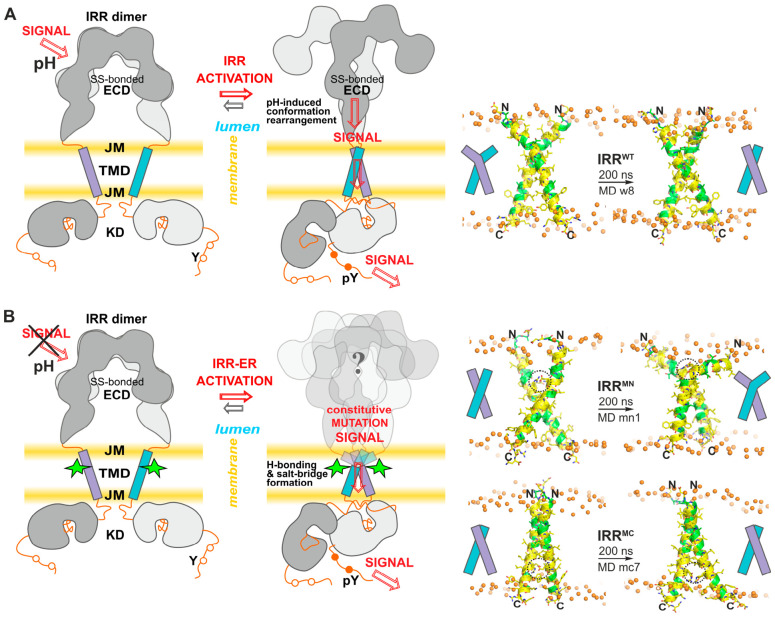
Schematic illustration of signal transduction across the cell membrane by the wild-type IRR and its ER-mutant forms. (**A**) In the inactive state at neutral pH values, the wild-type IRR adopts a Λ-shaped conformation via its disulfide-linked extracellular domain (ECD), while TM-domain (TMD) helices and kinase domains (KDs) remain largely separated. The pH increase induces conformational changes in the ECD that bring their C-termini closer together, resulting in dimerization of TM helices via the N-terminal weakly polar pattern. The receptor switching to an active T-shaped conformation involves rearrangements and proper dimerization of the intracellular juxtamembrane (JM) regions and kinase domains (KDs), which enables autophosphorylation of the target tyrosine residues (pY) and subsequent stimulation of downstream signaling cascades. On the right, a representative structure of the wild-type IRR TM domain dimerized via the N-terminal motif into a left-handed α-helical bundle is shown. Two ribbon structures before and after 200 ns unrestrained MD simulation (w8) are presented. (**B**) The N-terminal ER mutation (V929E-G930R) promotes N-terminal dimerization of the TM domains, thereby locking the receptor in a constitutively active conformation that is insensitive to alkaline pH. The C-terminal ER mutation (A938E-A939R) is also capable of inducing dimerization of the TM domain in the active N-terminal mode, favoring an active IRR conformation that is weakly affected by alkaline pH. Currently, the ECD conformation of the mutant receptors remains unknown. According to structural-dynamic NMR studies [15], the IRR TM helices exhibit bending plasticity at the flexible P-hinge (near intramembrane proline P^928^), which allows for compensation for the large distance between the ECD C-termini inherent in the inactive Λ-shaped conformation. Alternatively, the ECD dimer may adopt the active Γ- or T-shaped conformations, accompanied by straightening of the TM helices. Representative dimeric structures (right- and left-handed α-helical bundles from MD simulations mn1 and mc7) of the IRR TM domain with the N- and C-terminal ER substitutions promoting TM-domain association in an active N-terminal mode are shown on the right. Dashed ovals mark highly polar ER mutations responsible for intermonomeric hydrogen bonding and salt-bridge formation, which can stabilize the active N-terminal dimerization mode of the mutant IRR TM domain.

The present work extends our earlier studies by providing new evidence for possible conformational transitions in RTKs of the insulin receptor family, specifically addressing whether TM-region interactions enhanced by pro-oncogenic polar mutations can induce receptor activation (Figure 4). Notably, we have shown that double-ER substitution (V929E-G930R) within the putative N-terminal dimerization motif of the IRR TM domain, similar to analogous HER2 oncogenic mutation [2], results in strong autophosphorylation of IRR at neutral pH values. Neither the wild-type form nor the C-terminal A938E-A939R mutant exhibits such pH-independent activation under neutral and mildly alkaline conditions (at pH values above 7.4), underscoring the specificity of this ER substitution and implying that the N-terminal GG4-like motif of the IRR TM domain is involved in functional dimerization during receptor activation. Importantly, both ER substitutions in the IRR TM domain induce phosphorylation of downstream signaling proteins IRS-1 and Erk, suggesting the formation of functionally active receptor dimers capable of initiating intracellular signaling.

The observed pH-dependent activation of IRR mutant forms with single-point mutations V929E and G930R also emphasizes the unique properties of the double-ER substitution (V929E-G930R). Based on molecular modeling of possible dimerization modes of the IRR TM domain, both N- and C-terminal ER substitutions can lead to strong electrostatic interactions within the lipid bilayer (Figure 4, Supplementary Figure S2). Structural analysis indicates that these mutations promote stabilization of the active IRR dimer via a network of hydrogen bonds and salt bridges formed by complementary glutamic acid and arginine residues across the dimer interface, revealing a precise molecular mechanism underlying constitutive receptor activation. Reduced phosphorylation of both mutant receptors upon treatment of cells with an acidic extracellular medium (pH 5.5–6.5) supports the existence of the interaction between glutamic acid and arginine residues across the dimerization interface, as partial protonation of glutamate at acidic pH would abolish the electrostatic attraction of their ionogenic groups. The slight shift in IRR pH sensitivity toward more alkaline values observed for the A939R single-point mutation may also be attributed to the aforementioned charge neutralization of the arginine side chain at higher pH values within the membrane environment.

## 4. Materials and Methods

### 4.1. Dimer Structure Prediction with PredDimer Algorithm

We used the PredDimer Web server (http://model.nmr.ru/preddimer, accessed from 9 June 2025, [16]) to construct preliminary models of dimeric states for TM domains of the IRR receptor (referred to as IRR^WT^) and two mutant variants with ER substitutions, i.e., V929E-G930R and A938E-A939R shifted to the N-terminus (IRR^MN^) or C-terminus (IRR^MC^), respectively (Table S1). To enhance conformational sampling, we used multiple calculations with varying amino acid sequences adding 1–2 residues to the N- or C-terminus, with 7 variants for each peptide in total, as shown in Table S2. We arranged the results by the increase in crossing angle between helical axes from negative (right-handed dimers) to positive (left-handed dimers) and added terminal charged residues that are crucial for normal transmembrane orientation in molecular dynamics (MD) simulations. Energy minimization by the steepest descent algorithm was applied to each structure using the GROMACS [20] package (2018 version) with Amber 14SB protein parameters [21]. Then, we clustered models based on root-mean-square deviation values calculated between coordinates of the corresponding Cα atoms, interaction interface similarity, and dimer geometry (intermonomer distance and crossing angle between TM helical axes).

### 4.2. MD Simulations in Explicit Lipid Bilayer

Representative dimer models from each cluster were inserted into a pre-equilibrated, hydrated palmitoyloleylphosphatidyl choline (POPC) bilayer composed of 200 lipid molecules and approximately 10,000 water molecules. Overlapping lipid and water molecules were removed, and ions were added to neutralize charges. We used the GROMACS [20] package (2018 version or higher) for simulations and data processing. The all-atom Amber14SB force field was used for protein [21] with S-lipid parameters for POPC [22] and the TIP3P water model [23]. Constructed solvated systems were subjected to energy minimization, MD relaxation with restrained protein, and unrestrained MD simulations to estimate the stability of the system over 200 ns long trajectories with a constant temperature of 315 K and pressure of 1 atm. Relaxation was done by a 10 ns MD simulation with restraints imposed on the protein that were released in a step-wise manner: first, we restrained all protein atoms for 3 ns and used a Berendsen barostat and V-rescale algorithms for fast equilibration, then switched to backbone restraints with a more precise Nose–Hoover thermostat and Parrinello–Rahman barostat. Production runs were performed without any restraints on protein or environment. Structural stability was estimated in terms of the root-mean-square deviation (RMSD) of backbone atom coordinates from the starting structure, changes in the secondary protein structure, and comparison of interaction interfaces before and after MD simulations, followed by the calculation of dimer geometry parameters (crossing angle and intermonomer distance).

### 4.3. Molecular Hydrophobicity Maps

The molecular hydrophobicity potential (MHP) approach [24] was used to assess the spatial surface properties of TM α-helices of various RTKs. The MHP constants for peptide atoms were assigned according to [25]; calculations of the molecular surfaces of peptides and their interpolation onto a cylinder were performed as described in [16]. Average MHP maps were generated using frame sets from 200 ns MD trajectories. Contact areas were detected by solvent-accessible surface calculations and marked on maps with contours. To match features calculated for different conformations (predicted, relaxed, or MD average) we used the fit of Cα coordinates of the L924-G941 fragment, which preserves the helical structure in most of the simulations.

### 4.4. IRR Receptor Mutagenesis

For the generation of constructions with point mutations in the TM domain of IRR, we used the megaprimer PCR approach as described in [12]. The following primers were used for generation of single and double mutations (see Table 1).

### 4.5. Cell Cultures, Transfections, and Treatments

HEK 293 cells were cultured in DMEM supplemented with 10% fetal bovine serum (Hyclone, Logan, UT, USA), 1% penicillin/streptomycin, and 2 mM L-glutamine. The HEK 293 cells, at a confluent density of about 50%, were transfected by cDNA of human IRR receptor and its mutant forms using GenJect39 (Molecta, Moscow, Russia) according to the manufacturer’s protocol. At 36 h following transfection, cells were washed with serum-free F-12 medium and incubated for 3 h in serum-free F-12 medium containing 1% penicillin/streptomycin in a CO_2_ incubator to reduce the background of phosphorylated cell proteins. The cells were further incubated in serum-free F-12 medium buffered with 50 mM Tris-HCl at different pH values in the range of 7.4 to 9.4 in small increments for 10 min at 37 °C. For acidic conditions, F-12 medium was adjusted with 50 mM MES buffer at a pH of 6.5 or 5.5. Then, cells were lysed with SDS-PAGE sample buffer (75 mM Tris-HCl pH 6.8, 1,5% SDS, 150 mM b-MeEtOH, and 15% Glycerol), separated by electrophoresis, and analyzed by Western blotting with antibodies to phosphorylated (anti-pIRR) and total forms of IRR (anti-C-hIRR). 

### 4.6. Antibodies and Immunoblotting

Rabbit anti-C-hIRR antibodies were raised against the human IRR C-terminal cytoplasmic domain (amino acids 945–1297) expressed in bacteria as a His-tagged protein. PCR fragment-encoding amino acids 945–1297 of IRR were cloned into the pET28a vector (Novagen) using *Nco*I and *Xho*I restriction sites. The anti-pIRR antibodies were raised against KLH-coupled peptide CGMTRDVpYETDpYpYRKGGKGL from the activation loop of IRR as described in [5].

The cell lysates were separated by electrophoresis in 8% SDS-polyacrylamide gel, followed by blotting onto ECL-grade nitrocellulose (GE healthcare, Chicago, IL, USA). The blots were probed with rabbit anti-pIRR, rabbit anti-C-hIRR, rabbit anti-IRS-1 (Cell Signaling, #2382, Danvers, MA, USA), rabbit anti-phospho-IRS-1 (Y895) (Cell Signaling, #3070), rabbit anti-pAKT (Ser473) (Cell Signaling #193H12), rabbit anti-pErk (Thr202/Tyr204) (Cell Signaling #D13.14.4E), mouse anti-β-actin (8H10D10) (Cell Signaling, #3700), and mouse anti-phosphotyrosine (Millipore, clone 9G10, Burlington, MA, USA) antibodies. Blots were blocked in 5% non-fat milk or 2% BSA (for phosphoprotein detection) in TBST buffer (10 mM Tris–HCl, pH 7.8, 150 mM NaCl, and 0.1% Tween 20), then incubated with primary antibodies. After incubation with horseradish peroxidase-conjugated anti-rabbit or anti-mouse secondary antibodies (Jackson ImmunoResearch, West Grove, PA, USA), immunoreactive bands were visualized by enhanced chemiluminescence (Pierce, WestPico, Appleton, WI, USA). For the quantitative analysis of Western blots, we used the Fusion Solo system (Vilber Lourmat, Collégien, France). The captured images were manually selected in rectangles and further analyzed by densitometry with Fusion software (FusionCapt Advance 16.09b, Vilber Lourmat, Marne-la-Vallée, France). Final calculations were made using GraphPad 6.0.1 software (GraphPad Software). The ratio of the integral density of the phosphorylated receptor (pIRR antibody signal) to the total receptor (IRR antibody signal) was plotted versus pH.

## 5. Conclusions

In summary, we can conclude that the IRR TM domain plays a pivotal role in receptor function. We have shown that site-directed mutagenesis of the IRR TM domain can switch the receptor to a constitutively active state that is insensitive or weakly affected by alkaline pH. The bioassay and modeling data also suggest that upon receptor activation and signal transduction across the cell membrane, the IRR TM helices dimerize via their N-terminal GG4-like motif. Thus, our findings offer a strategy of using single and double amino acid substitutions for targeted modulation of functional properties of the receptor, including its pH-dependent activity. Furthermore, such ER mutant-mediated control of receptor signaling could be extended to other insulin receptor family members and RTKs, proving useful in developing new targeted therapies for human diseases.

## Figures and Tables

**Table 1:** The structure of the oligonucleotide primers used to obtain mutant forms of the IRR receptor.

Mutant	Primer (5′–3′)
IRR_V929E_G930R	AACGATGAGCAGCGTGAGCCGCTCAGGGGTGGCAGTGAGGAG
IRR_V929E	GATGAGCAGCGTGAGCCCCTCAGGGGTGGCAGTGAGGAG
IRR_G930R	AACGATGAGCAGCGTGAGCCGCACAGGGGTGGCAGTGAGG
IRR_A938E_A939R	TTGCCGTAGAAGAAACCAAGGCGCTCAAGAACGATGAGCAGCGTGA
IRR_A938E	GCCGTAGAAGAAACCAAGGGCCTCAAGAACGATGAGCAGCGTGA
IRR_A939R	TTGCCGTAGAAGAAACCAAGGCGAGCAAGAACGATGAGCAGCG

## Data Availability

The original contributions presented in this study are included in the article/Supplementary Material. Further inquiries can be directed to the corresponding authors.

## Supplementary Materials

The following supporting information can be downloaded at: https://www.mdpi.com/article/10.3390/ijms27104364/s1.
