# Supplementary material for: Insulin Receptor-Related Receptor Activation by Artificial Double-ER Mutations in the Transmembrane Domain
Source: Int J Mol Sci. 2026 May 14;27(10):4364. doi: 10.3390/ijms27104364 (PMC13207528; doi:10.3390/ijms27104364)
Supplement: Supplementary file 1 [file ijms-27-04364-s001.zip › IRRmut_ijms_suppl.pdf]

Supplementary Materials

Insulin Receptor-Related Receptor Activation by Artificial Double-ER Mutations in the Transmembrane Domain

Oxana V. Serova[1,†], Alina A. Gavrilenkova[1,2,†], Andrey S. Kuznetsov[1], Alexander S. Goryashchenko[1], Alexandra R. Agisheva[1], Yaroslav V. Bershatsky[1,3], Vladislav A. Lushpa[1,2], Olga T. Zangieva[4], Mikhail S. Karbyshev[5], Andrei S. Gerasimov[2,3], Ivan S. Okhrimenko[2], Roman G. Efremov[1,3], Igor E. Deyev[1,2,*], Eduard V. Bocharov[1,2,*]

[1] Shemyakin–Ovchinnikov Institute of Bioorganic Chemistry, Russian Academy of Sciences, Miklukho-Maklaya Str. 16/10, 117997 Moscow, Russia; oxana.serova@gmail.com (O.V.S.); alycat1008@gmail.com (A.A.G.); andrej.kuznecov@phystech.edu (A.S.K.); asgoryash@gmail.com (A.S.G.); alexagisheva@gmail.com (A.R.A.); bershackyjaroslav@gmail.com (Y.V.B.); lushpa1696@gmail.com (V.A.L.); r-efremov@yandex.ru (R.G.E.)

[2] Moscow Center of Advanced Studies, Kulakova Str. 20/1, 123592 Moscow, Russia; asgerasimoff@mail.ru (A.S.G.); i.s.okhrimenko@yandex.ru (I.S.O.)

[3] Scientific Research Institute for Systems Biology and Medicine, Nauchny Proezd 18, 117246 Moscow, Russia

[4] National Medical and Surgical Center named after N. I. Pirogov, Nizhnyaya Pervomayskaya str. 70, 105203 Moscow, Russia; olga.dok.oz@gmail.com

[5] Research Division, Moscow Polytechnic University, Bolshaya Semyonovskaya ul. 38, Moscow 107023, Russia; mikhail_karbyshev@hotmail.com

* Correspondence: deyevie@ibch.ru (I.E.D.); bon@nmr.ru (E.V.B.)

† These authors have contributed equally to this work.

Supplementary table content:

Table S1. Transmembrane peptide sequences used for the IRR TM domain dimer structure predictions

Table S2. TM peptide sequences used for MD simulations of the IRR TM domain dimer in explicit POPC bilayer

Table S3. Representative dimeric structures of the IRR TM domain after energy minimization and clustering with their parameters

Table S4. Average parameters of the IRR TM domain dimer calculated from MD trajectories

Figure S1. MD simulations of the TM domain dimerization interfaces of wild-type and ER-mutant IRR

Figure S2. MD simulations of the TM domain dimer conformations of wild-type and ER-mutant IRR

Table S1. Transmembrane peptide sequences used for the IRR TM domain dimer structure predictions

Peptide	Amino acid sequence used in PredDimer
IRR^{WT}	^{922}VLLTATPVGLTLLIVLAALGFFY^{944}
	^{922}VLLTATPVGLTLLIVLAALGFFYG^{945}
	^{922}VLLTATPVGLTLLIVLAALGFFYGK^{946}
	^{921}HVLLTATPVGLTLLIVLAALGFFY^{944}
	^{921}HVLLTATPVGLTLLIVLAALGFFYG^{945}
	^{921}HVLLTATPVGLTLLIVLAALGFFYGK^{946}
	^{920}LHVLLTATPVGLTLLIVLAALGFFYGKK^{947}
IRR^{MN}	^{922}VLLTATP**ER**LTLLIVLAALGFFY^{944}
	^{922}VLLTATP**ER**LTLLIVLAALGFFYG^{945}
	^{922}VLLTATP**ER**LTLLIVLAALGFFYGK^{946}
	^{921}HVLLTATP**ER**LTLLIVLAALGFFY^{944}
	^{921}HVLLTATP**ER**LTLLIVLAALGFFYG^{945}
	^{921}HVLLTATP**ER**LTLLIVLAALGFFYGK^{946}
	^{920}LHVLLTATP**ER**LTLLIVLAALGFFYGKK^{947}
IRR^{MC}	^{922}VLLTATPVGLTLLIVL**ER**LGFFY^{944}
	^{922}VLLTATPVGLTLLIVL**ER**LGFFYG^{945}
	^{922}VLLTATPVGLTLLIVL**ER**LGFFYGK^{946}
	^{921}HVLLTATPVGLTLLIVL**ER**LGFFY^{944}
	^{921}HVLLTATPVGLTLLIVL**ER**LGFFYG^{945}
	^{921}HVLLTATPVGLTLLIVL**ER**LGFFYGK^{946}

Table S2. TM peptide sequences used for MD simulations of the IRR TM domain dimer in explicit POPC bilayer

Peptide	Amino acid sequence
IRR^{WT}	^{916}DAGGLHVLLTATPVGLTLLIVLAALGFFYGKKRN^{949}
IRR^{MN}	^{916}DAGGLHVLLTATP**ER**LTLLIVLAALGFFYGKKRN^{949}
IRR^{MC}	^{916}DAGGLHVLLTATPVGLTLLIVL**ER**LGFFYGKKRN^{949}

Table S3. Representative dimeric structures of the IRR TM domain after energy minimization and clustering with their parameters

Peptide	Cluster	Number of structures	Residues on the dimer interface	Cross angle, deg	Distance, nm
	w1	5	_________________LL__LA_LG__Y_____	-48	0.73
	w2	5	_______L______G__LL__LA__GF_______	-36	0.89
	w3	9	_____H__LT__PV__T___V___L__FY_KK__	-31	0.91
IRR^{WT}	w4	7	____L__L__AT__G__LL__L___GF_YG__R_	-3	0.88
	w5	5	_____H__LT__P___T__I___A__FF_GK___	4	0.91
	w6	10	D__G__VL_____V___L__V___L__FY_KK_N	18	0.93
	w7	6	________________T__I______F_______	41	1.00
	w8	7	__________AT__G__LL__L______Y_____	51	0.79
	mn1	7	______________R__LL__L____F_______	-50	0.97
	mn3	2	________________T__IV___L__FY_____	-50	0.87
	mn4	3	D__G___L_____ER__LL__L___G___G__R_	-14	0.95
IRR^{MN}	mn6	15	__GG_HV__T______T___V______F__K___	5	0.99
	mn6a	3	DAGGL_V___A__E______V___L__FY__KR_	7	1.15
	mn7	7	______V__T______T__IV__A__FF______	40	0.90
	mn9	3	_____________E__TL__V______F______	46	0.86
	mc1	2	_________________LL__LE__G__Y_____	-53	0.77
	mc1a	4	__________________L__LE__GF_______	-51	0.90
	mc3	4	_____H___T___V__TL__VL__L___Y_____	-48	0.85
	mc2	7	_______L__AT__GL__L___E__GF_______	-28	0.96
IRR^{MC}	mc4	4	____L__L__AT__G__LL__LE__G__YG__R_	3	0.90
	mc6	4	DAGGLHV______V___L__V__RL__FY__K__	5	1.08
	mc7	16	_____H___T__P___T__I__ER__FF______	21	0.91
	mc7a	2	__G___V__T___V__T__IV__R______K___	28	0.96
	mc8	5	__________AT__G__LL__L__L___Y_____	42	0.81

Table S4. Average parameters of the IRR TM domain dimer calculated from MD trajectories

Peptide	Structure	RMSD, nm	Residues on the dimer interface	Cross angle, deg	Distance, nm
IRR^{WT}	w1	0.46±0.11	____________________L___GF_YGK___<br>________________L___L__LG__Y___R_	-51 ± 32	0.83 ± 0.45
	w2	0.42±0.02	___G__V_______G___L__LA__G___G____<br>____L__L_________L___A___F_____R_	-32 ± 4	0.83 ± 0.08
	w3	0.42±0.09	___________PV__T___V___L_________<br>____________V__T___V___L___Y_____	-30 ± 6	1.08 ± 0.20
	w4	0.33±0.09	____L__LL__T______L______GF__G__R_<br>____L__L___T___L__L__LA__G__YG____	12 ± 6	0.90 ± 0.12
	w5	0.44±0.07	_______________T__I___A__FF_GK___<br>_______________T__I___A__FF_GK___	21 ± 8	0.96 ± 0.21
	w6	0.33±0.04	D_GG__V_________L__V___L__FY_____<br>___G__V______V__L__V___L__F______	28 ± 5	0.91 ± 0.09
	w7	0.45±0.05	_______L___P__L___I_____________<br>_______________T__I___A__F_______	30 ± 5	1.04 ± 0.10
	w8	0.36±0.05	____L__L______G__L___L__L_________<br>_______L______G__L___L______Y_____	42 ± 6	0.85 ± 0.10
IRR^{MN}	mn1	0.58±0.09	_____________R___L_______________<br>_____________R__LL_______________	-54 ± 8	1.29 ± 0.12
	mn3	0.57±0.39	_______________T__IV___L__F______<br>_______________T__IV__AL__F______	-46 ± 23	1.09 ± 0.90
	mn4	0.42±0.04	___G__VL_____ER___L__L____________<br>DA____V__T___ER__L___L____________	-30 ± 5	1.15 ± 0.06
	mn6	0.57±0.05	D_G__HV__T___E__T__IV_____F_______<br>_____H__LT__P__LT__I______F_______	23 ± 8	0.91 ± 0.10
	mn6a	0.41±0.04	DAGG_HV______E___L__V___L__F______<br>DAGG_HV______ER__L__V___L___Y_____	29 ± 4	1.05 ± 0.07
	mn7	0.83±0.07	_______________T__IV__A__FF______<br>_______________T__IV__A__FF__K___	44 ± 10	1.06 ± 0.22
	mn9	1.10±0.83	_______________TL__V_____FF______<br>____________E___L__V___L__F______	29 ± 28	1.82 ± 1.02
IRR^{MC}	mc1	0.42±0.07	____________________LE_LGF_Y___R_<br>________________L___LE_____Y___R_	-59 ± 16	0.90 ± 0.12
	mc1a	0.36±0.05	_A_______________L___E___________<br>_____________G__LL__L___________	-52 ± 5	0.86 ± 0.13
	mc3	0.53±0.09	_____HV__T___V______________Y__KR_<br>_____H___T__PV__T___V______F___K__	-16 ± 9	1.15 ± 0.07
	mc2	0.48±0.05	____L____________________________<br>_______L___T______L_______________	-28 ± 12	1.20 ± 0.15
	mc4	0.65±0.13	________________L___LE__G__YG____<br>_________________L__LE__G___GK_R_	-29 ± 8	0.95 ± 0.16
	mc6	0.47±0.04	D___________________V___L__FY__K__<br>__G_________________V___L__FY_____	28 ± 5	0.95 ± 0.07
	mc7	0.39±0.03	_________T__P___T__I__ER__F_______<br>_____H___T__P___T__I__ER__F_______	30 ± 5	1.02 ± 0.04
	mc7a	0.52±0.05	D_GG__V___A__V__TL_____R__________<br>D_____V__T___V__T___V__R__________	29 ± 5	1.03 ± 0.05
	mc8	0.39±0.06	_______L______G__L___L______Y_____<br>_______L___T__G__L___L__L_________	44 ± 4	0.74 ± 0.06

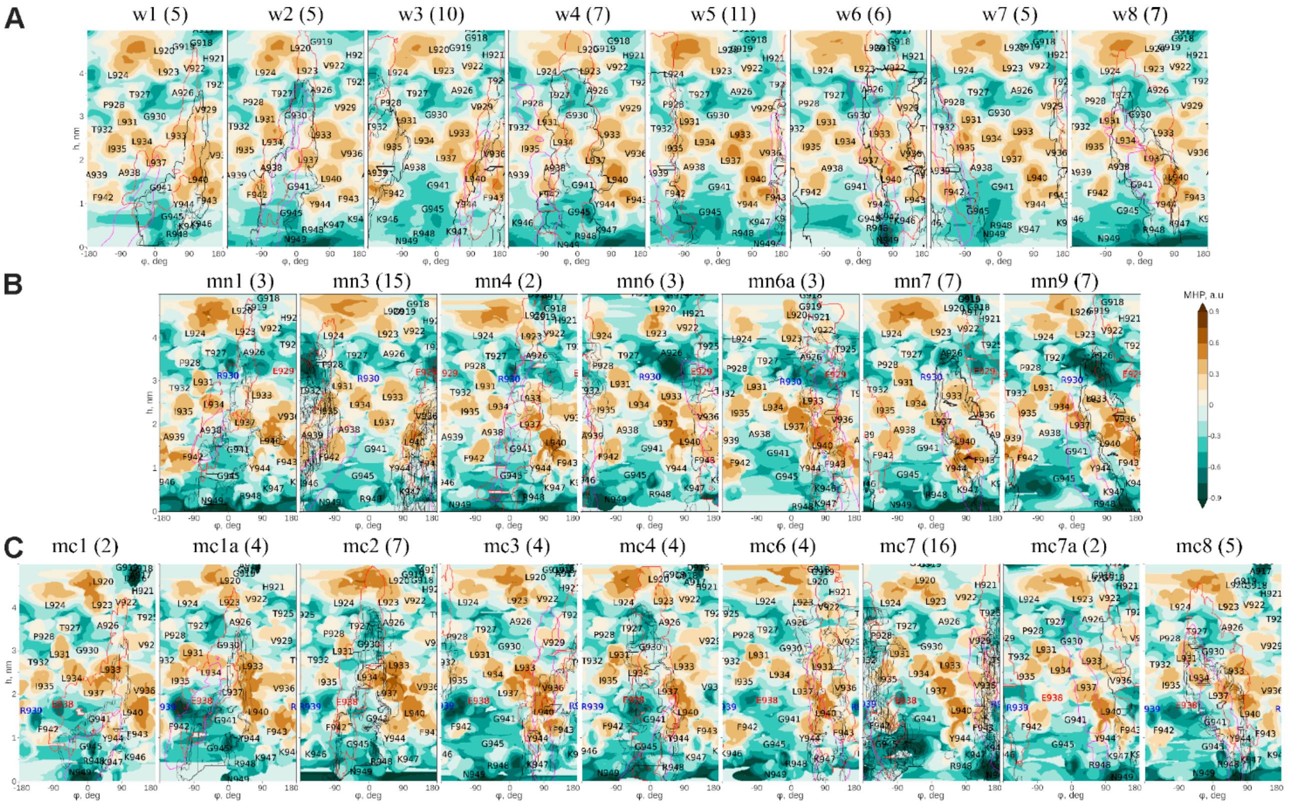

Figure S1. MD simulations of the TM domain dimerization interfaces of wild-type and ER-mutant IRR.
Molecular hydrophobicity potential (MHP) distribution maps for possible TM domain dimer conformations of IRR^{WT} (**A**), IRR^{MN} with the N-terminal double mutation V929E-G930R (**B**), and IRR^{MC} with the C-terminal double mutation A938E-A939R (**C**) are presented. The cylindrical projection of the MHP distribution on the IRR TM helix surface is used. Axis values correspond to the rotation angle around the helical axis (φ) and the distance along the latter (h), respectively. 2D maps are colored according to MHP arbitrary units [1] from blue (hydrophilic areas) to brown (hydrophobic ones). The maps represent MD-relaxed structures obtained from PredDimer [2] cluster leaders with weak restraints on protein backbone atoms. Contact area between monomers in the corresponding TM dimers is marked by contours: black contours represent all cluster structures, red line is for MD-relaxed structure, and violet is an average of the last 50 ns part of 200-ns unrestrained MD simulation. Residual marks correspond to average positions of their C_{α} atoms during MD relaxation. The residual marks of E and R corresponding to N- and C-terminal substitutions V659E-G660R and A938E-A939R are highlighted by red and blue.

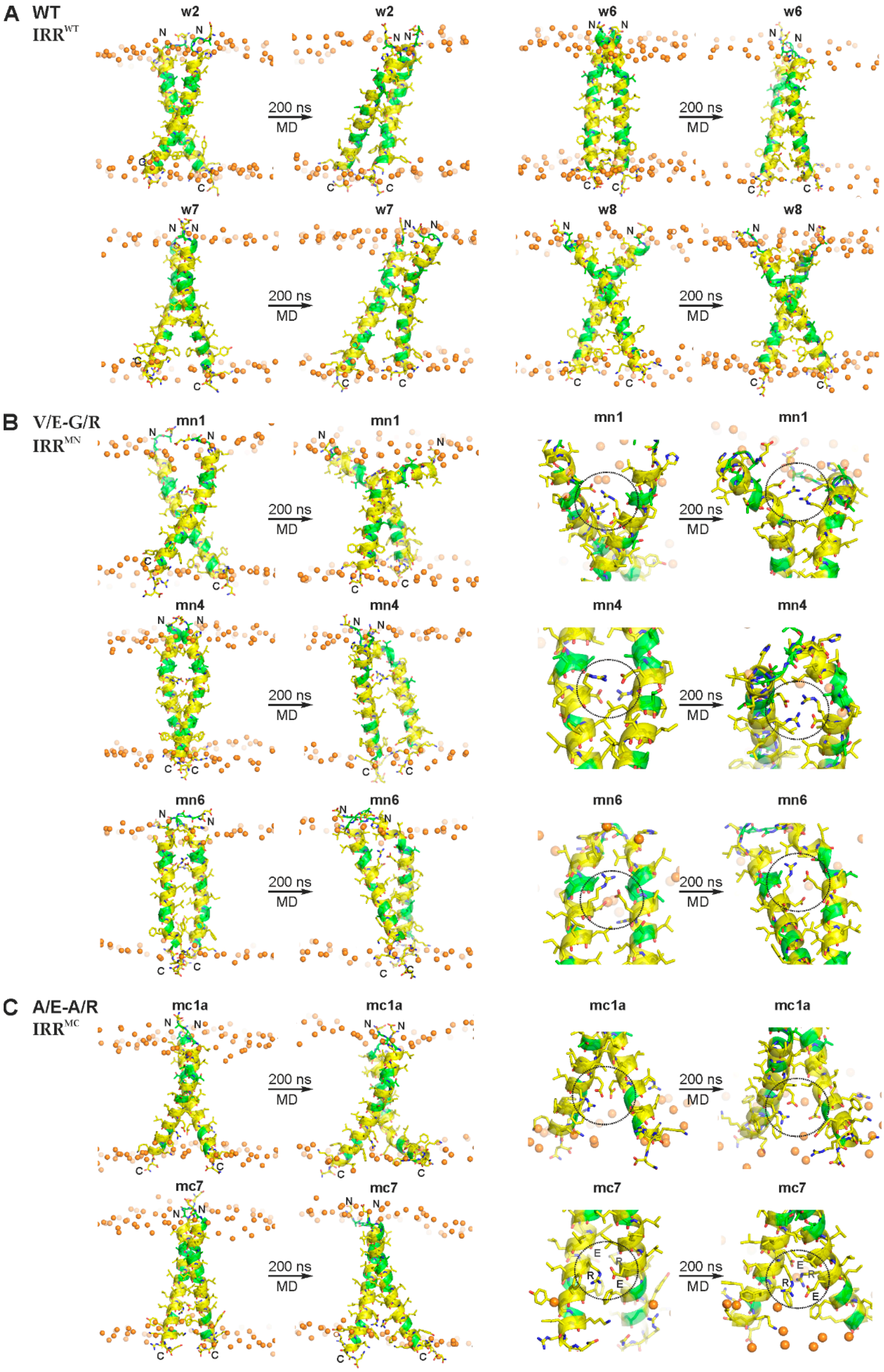

Figure S2. MD simulations of the TM domain dimer conformations of wild-type and ER-mutant IRR. Representative dimeric structures (right- and left-handed α-helical bundles) of wild-type IRR TM domain, IRR^{WT} (**A**), and of the IRR TM domain with N- and C-terminal double mutations V929E-G930R, IRR^{MN} (**B**), and A938E-A939R, IRR^{MC} (**C**), before and after 200-ns unrestrained MD simulation are presented. In the zoomed ribbon structures of the right panels in (**B**) and (**C**), dashed ovals mark highly polar ER mutations responsible for intermonomeric hydrogen bonding and salt bridge formation, which can stabilize the active N-terminal dimerization mode of the mutant IRR TM domain, thereby locking the receptor in a constitutively active conformation that is fully insensitive to or weakly responsive to alkaline pH.

References

1. Wildman, S.A.; Crippen, G.M. Prediction of Physicochemical Parameters by Atomic Contributions. *J. Chem. Inf. Comput. Sci.* **1999**, *39*, 868–873, doi:10.1021/ci990307l.
2. Polyansky, A.A.; Chugunov, A.O.; Volynsky, P.E.; Krylov, N.A.; Nolde, D.E.; Efremov, R.G. PREDDIMER: A Web Server for Prediction of Transmembrane Helical Dimers. *Bioinforma. Oxf. Engl.* **2014**, *30*, 889–890, doi:10.1093/bioinformatics/btt645.
