# Supplementary figures and images for: Insulin Receptor-Related Receptor Activation by Artificial Double-ER Mutations in the Transmembrane Domain
Source: Int J Mol Sci. 2026 May 14;27(10):4364. doi: 10.3390/ijms27104364 (PMC13207528; doi:10.3390/ijms27104364)

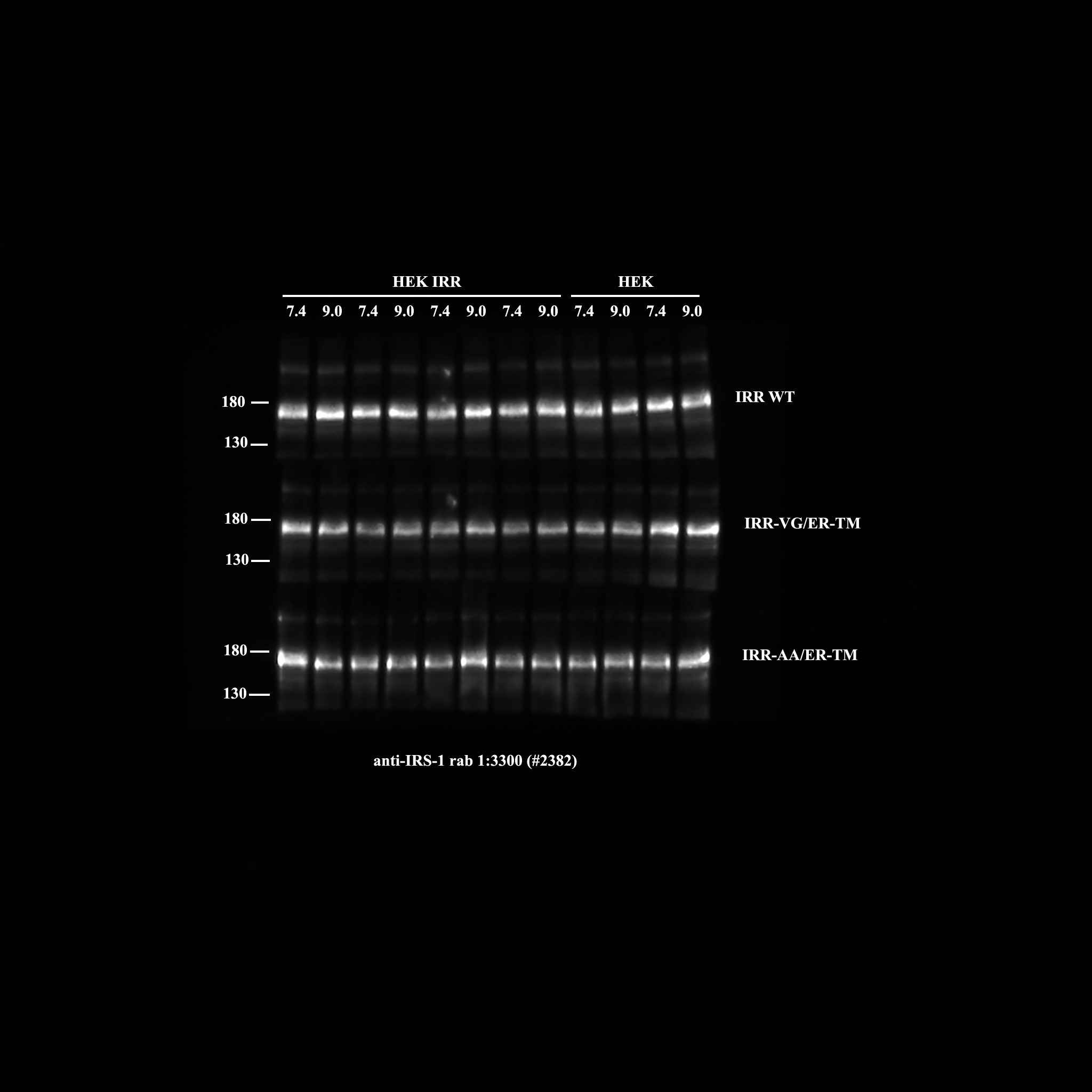

Supplement: Supplementary file 1 [file ijms-27-04364-s001.zip › IRS-1_blot.jpg]

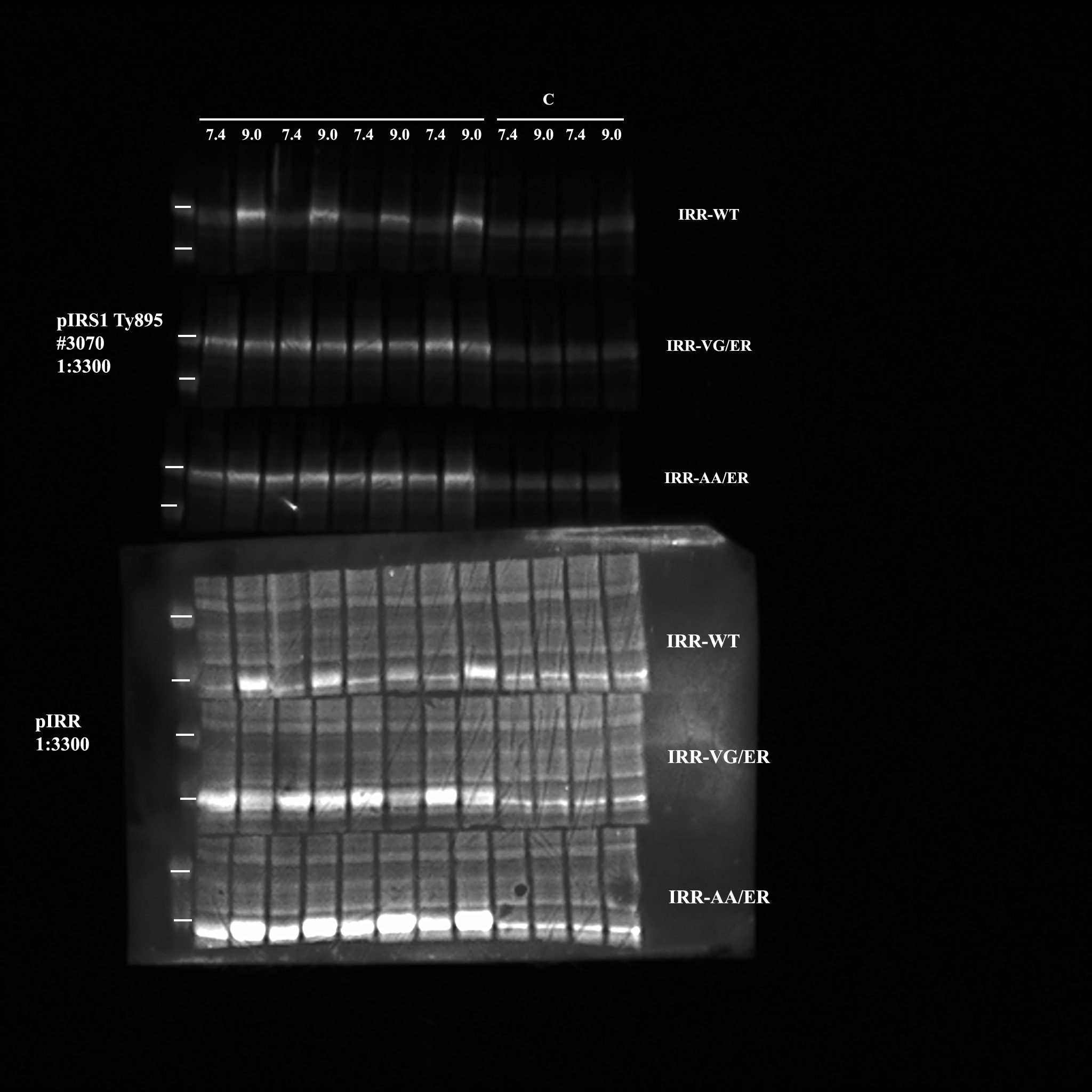

Supplement: Supplementary file 1 [file ijms-27-04364-s001.zip › pIRS1_pIRR_blots.jpg]
